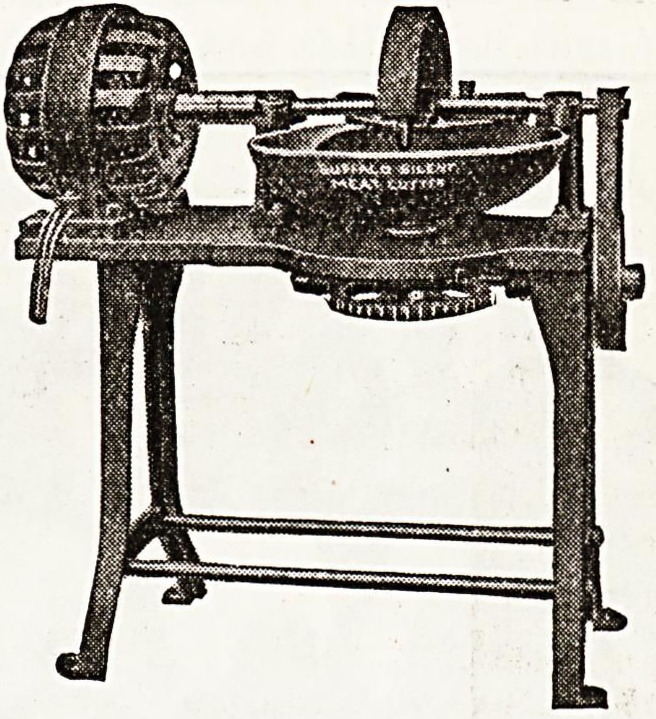# Institutional Needs

**Published:** 1913-11-08

**Authors:** 


### Institutional Needs.

### NEW MEAT AND VEGETABLE CHOPPER.

John E. Smith's Sons Company, of Buffalo, New York, U.S.A., have placed on the market a remarkable sanitary meat, food, and vegetable chopper, both of the hand and motor driven variety. No. 16 outfit, of which an illustration appears in this issue, is worth a somewhat detailed description. This machine has a capacity for 12 lb. of meat in five minutes, and cooked meat and vegetables in two minutes; consequently the makers may be understood in claiming that an institution with 500 people to feed would find this chopper large enough for its purpose. Its weight is 400 lb., and its price is from

$160. The floor space occupied is 36 in. wide by 24 in. deep. The great point made by the makers is that it is no grinder or masher, but that it cuts raw meat finely, and also vegetables. The Johns Hopkins Hospital, Baltimore, and the Montreal General Hospital are among the best of American and Canadian institutions which employ the "Buffalo," and the numerous testimonials it has received testify to its value. The officer commanding the Army and Navy General Hospital is quoted as saying: "The machine is doing all you claim. It does not mash the food, saves juice, cuts and mixes at the same time, is easily kept clean, and is entirely noiseless." As a labour-saving device it needs no testimony.

## Figures and Tables

**Figure f1:**